# Comparison of Bayesian vs Frequentist Adaptive Trial Design in the Stroke Hyperglycemia Insulin Network Effort Trial

**DOI:** 10.1001/jamanetworkopen.2022.11616

**Published:** 2022-05-11

**Authors:** Kristine Broglio, William J. Meurer, Valerie Durkalski, Qi Pauls, Jason Connor, Donald Berry, Roger J. Lewis, Karen C. Johnston, William G. Barsan

**Affiliations:** 1 AstraZeneca US, Gaithersburg, Maryland; 2 Berry Consultants LLC, Austin, Texas; 3 Department of Emergency Medicine, University of Michigan, Ann Arbor; 4 Department of Neurology, University of Michigan, Ann Arbor; 5 Stroke Program, University of Michigan, Ann Arbor; 6 Department of Public Health Sciences, Medical University of South Carolina, Charleston; 7 ConfluenceStat LLC, Cooper City, Florida; 8 Department of Medical Education, University of Central Florida College of Medicine, Orlando; 9 Department of Emergency Medicine, Harbor-UCLA Medical Center, Torrance, California; 10 Department of Emergency Medicine, David Geffen School of Medicine at UCLA, Los Angeles, California; 11 Department of Neurology, University of Virginia, Charlottesville

## Abstract

**Importance:**

Bayesian adaptive trial design has the potential to create more efficient clinical trials. However, a barrier to the uptake of bayesian adaptive designs for confirmatory trials is limited experience with how they may perform compared with a frequentist design.

**Objective:**

To compare the performance of a bayesian and a frequentist adaptive clinical trial design.

**Design, Setting, and Participants:**

This prospective cohort study compared 2 trial designs for a completed multicenter acute stroke trial conducted within a National Institutes of Health neurologic emergencies clinical trials network, with individual patient-level data, including the timing and order of enrollments and outcome ascertainment, from 1151 patients with acute stroke and hyperglycemia randomized to receive intensive or standard insulin therapy. The implemented frequentist design had group sequential boundaries for efficacy and futility interim analyses at 90 days after randomization for 500, 700, 900, and 1100 patients. The bayesian alternative used predictive probability of trial success to govern early termination for efficacy and futility with a first interim analysis at 500 randomized patients and subsequent interims after every 100 randomizations.

**Main Outcomes and Measures:**

The main outcome was the sample size at end of study, which was defined as the sample size at which each of the studies stopped accrual of patients.

**Results:**

Data were collected from 1151 patients. As conducted, the frequentist design passed the futility boundary after 936 participants were randomized. Using the same sequence and timing of randomization and outcome data, the bayesian alternative crossed the futility boundary approximately 3 months earlier after 800 participants were randomized.

**Conclusions and Relevance:**

Both trial designs stopped for futility before reaching the planned maximum sample size. In both cases, the clinical community and patients would benefit from learning the answer to the trial’s primary question earlier. The common feature across the 2 designs was frequent interim analyses to stop early for efficacy or for futility. Differences between how these analyses were implemented between the 2 trials resulted in the differences in early stopping.

## Key Points

**Question:**

How would an entirely prespecified alternative bayesian adaptive design perform compared with a frequentist clinical trial of glycemic control in acute ischemic stroke?

**Findings:**

In this cohort study comparing 2 trial designs for a randomized clinical trial, the bayesian design met the criteria for futility at 800 enrollments, whereas the implemented frequentist design did not meet the futility criteria until 936 enrollments.

**Meaning:**

These findings suggest that the trial potentially could have reached a similar conclusion earlier with the bayesian alternative design.

## Teaser

This cohort study compares the use of a bayesian vs frequentist trial design in the Stroke Hyperglycemia Insulin Network Effort trial.

## Introduction

Adaptive clinical trials use interim analyses of the accumulating trial data to make prospectively defined changes.^1^ These changes guide the use of the remaining trial resources to most efficiently address the trial’s primary scientific question. Two common adaptive design features in confirmatory trials are sample size re-estimation and early stopping.^1^ These 2 design features aim to align the total study size with the number of participants needed to answer the question. Both frequentist and bayesian approaches to sample size re-estimation and early stopping have been used in modern confirmatory trials. Additional innovative adaptive design features are increasingly feasible with advances in statistics and clinical trial operations. Bayesian statistical methods have contributed to this recent innovation because they more intuitively make use of different sources of information, such as intermediate and final end point assessments, and provide probability statements, which can be aligned with scientific priorities.^2^

Despite the potential of bayesian methods and adaptive clinical trial designs, there has been slow uptake of innovative design features in confirmatory phase trials and particularly in academic-led research efforts.^3^ To address this limitation, the US Food and Drug Administration (FDA) and the Office of the Director of the National Institutes of Health (NIH) launched the Advancing Regulatory Science initiative. One project funded under this program was a public-private partnership called the Adaptive Designs Accelerating Promising Trials Into Treatments (ADAPT-IT) project.^4^ In ADAPT-IT, academics from an NIH-funded neurologic emergency clinical trials network collaborated with other scientists with the objectives of designing bayesian adaptive confirmatory-phase neurologic emergency clinical trials and using mixed methods to gain insights into the process.^5^

The projects from ADAPT-IT are described elsewhere.^6,7,8^ The investigators planning the Stroke Hyperglycemia Insulin Network Effort (SHINE) trial^9^ were the first to participate in ADAPT-IT. A frequentist group sequential trial design with a blinded sample size re-estimation had been developed and funded, and that trial design was retained and executed. However, the ADAPT-IT process resulted in a second, fully specified, alternative adaptive design that was fully bayesian. The bayesian adaptive design used more frequent interim analyses to assess the trial for early stopping. Both designs were fully and rigorously simulated to determine their performance across a variety of realistic scenarios for how data may accumulate during the trial and designed to offer similar power with the expected 7% treatment effect. The alternative design was published along with a prospectively defined plan to collect the data that would have been necessary to conduct its analysis plan to compare the execution results of the 2 designs.^10^ Therefore, during the conduct of the SHINE trial, data snapshots were frozen whenever an interim analysis for the alternative bayesian design would have occurred. This has now allowed the alternative bayesian design to be virtually executed, using the actual trial data, exactly as would have happened had the bayesian design been selected for implementation.

One of the barriers to the uptake of bayesian adaptive designs for confirmatory trials is relative lack of experience with how they may perform and compare, in practice, to a frequentist design. The SHINE trial is a rare opportunity to compare more commonly used trial features (blinded sample size re-estimation and boundary-based rules for efficacy or futility stopping) to a counterfactual bayesian design, both prospectively designed by the same lead investigators around the same primary scientific question and goals. Only a few published examples exist of randomized clinical trials that have been retrospectively reconsidered with alternative trial designs,^11,12,13^ and we are unaware of any reports based on a predefined alternative. In this study, we report the results of the virtually executed bayesian alternative design of the SHINE trial.

## Methods

The SHINE trial was a randomized clinical trial that investigated intensive glucose control vs standard of care for patients with hyperglycemia who had a stroke.^14^ The primary efficacy end point was a dichotomous assessment of a favorable neurologic outcome at 90 days. A favorable outcome for a patient was defined based on a sliding dichotomy of the modified Rankin scale, dependent on baseline stroke severity.^15,16^ The maximum sample size was 1400 patients, which provided 80% power to detect a clinically meaningful improvement in the proportion of patients achieving favorable outcomes from 25% on the standard of care arm to 32% for the intensive glucose control arm based on a 2-sided frequentist test at the 5% level. The current cohort study was conducted with a SHINE trial data set that was free of personal identifiers. The data coordinating center did not have access to trial participant identities, and the investigators could not link the data to individuals. This was considered a nonregulated activity by the University of Michigan Institutional Review Board. No reporting guideline is relevant to concurrent alternative analyses of clinical trials, but the original trial followed Consolidated Standards of Reporting Trials (CONSORT) reporting guideline.

### SHINE Trial

The SHINE trial was executed using a group sequential trial design with 2-sided efficacy and futility stopping boundaries along with response-adaptive randomization. The stopping boundaries were defined using the Gamma family spending functions and closely resembled an O’Brien-Fleming boundary.^17^ Futility boundaries were considered nonbinding; therefore, if a futility boundary was crossed, the trial could be continued without penalty. Interim analyses were planned when 500, 700, 900, and 1100 consecutively randomized patients had reached the 90-day follow-up period. Multiple imputation was used to address missing primary outcome data for all interim and final analyses. A blinded sample size re-estimation was planned to occur before the first interim analysis. The blinded sample size re-estimation could increase the trial’s maximum sample size if the proportion of patients experiencing a favorable outcome was higher than expected. The full design is specified in the SHINE trial protocol and the statistical analysis plan, which are available as supplements to the main trial publication.^9^

### SHINE Alternative Design

The alternative trial design was fully bayesian in nature and developed to determine whether a bayesian approach could address the same clinical question but do so more efficiently. The alternative design was constrained to have the same primary end point, sample size, overall type I error rate, and power as the SHINE trial because aligning these design parameters was perceived to create a fair comparison between the approaches.

The alternative design was a bayesian adaptive Goldilocks trial design.^18^ The sample size re-estimation and early stopping included in the frequentist SHINE trial are focused on the goal of rightsizing the trial in response to the observed trial data. Similarly, the Goldilocks design is a sample size selection approach that aims to rightsize the number of patients randomized between a prespecified minimum and maximum in response to the observed trial data. This design included more frequent interim analyses, with the first interim analysis scheduled to occur after 500 patients were randomized and subsequent interims planned after every additional 100 patients were randomized. Trials using this approach have been used in the support of multiple regulatory approvals by the FDA.^19,20,21,22,23^

With the interim analyses timed according to the number of patients randomized, not all patients included in the interim analyses would have known 90-day primary end point outcomes. However, patients in the trial were also being assessed for a favorable neurologic outcome at 6 weeks, and so the bayesian design made use of that earlier information with a longitudinal model that linked the 6-week and 90-day outcomes. At each interim analysis, patients with 6-week and 90-day outcomes informed a longitudinal model of how the 6-week outcome may predict the 90-day outcome. On the basis of this currently observed association, as well as the corresponding uncertainty in this association, at each interim analysis, patients with a 6-week outcome but who had not yet completed follow-up for the 90-day outcome were included in the interim analysis with their 90-day outcome imputed via this longitudinal model. Patients without a 6-week or 90-day outcome contributed no outcome information at the interim analyses.

At each interim analysis, 2 predictive probabilities of trial success are calculated.^24^ The first is the predictive probability of trial success with the currently randomized patients. If this predictive probability is high, accrual may be stopped for early success. In this case, all randomized patients would complete follow-up for their primary end point, and the final analysis is then conducted on that complete data set. Therefore, a predictive distribution is used to make an early stopping decision to account for the additional data that will accumulate between the time accrual is stopped and the final analysis is conducted. The rationale is that if the currently randomized sample size provides sufficient power for the treatment effect being observed in the trial, then additional trial resources are not required. The second predictive probability is the predictive probability of trial success at the maximum sample size of 1400 patients. If this value is low, the trial may be stopped early for futility. The rationale in this case is that the maximum amount of trial resources would still not provide an adequate chance of trial success, and as such, the trial should end based on an inability to successfully address its primary objective. For the SHINE trial, the predictive probability at the current sample required to stop accrual early for success was set at 99%, and the predictive probability at the maximum sample size required to stop the trial early for futility was set at 5%. These values were selected to minimize the probability that the trial would stop accrual for predicted success and yet fail to achieve success at the final analysis with complete follow-up of all enrolled patients and to minimize the reduction in trial power because of the probability that the trial would stop early for futility incorrectly. The final analysis was a bayesian posterior probability of superiority. To account for the multiple interim analyses, the final critical value required for statistical significance was set at a bayesian posterior probability of superiority of at least 97.9%. This value was selected to control the overall 1-sided type I error of the trial to less than 2.5%. Thus, these futility rules are a binding futility stop. All prior distributions were noninformative. This design and its operating characteristics are completely described elsewhere.^10^

### Statistical Analysis

This study is a preplanned secondary analysis of the SHINE trial. The statistical and data management team for the SHINE trial preserved snapshots of the accumulating enrollment and outcome data consistent with the timing of interim analyses specified in the alternative bayesian design. After the SHINE trial was complete, these data sets were used to virtually conduct the interim analyses prespecified in the alternative design. The comparison of the trial executions was prespecified in the article by Connor et al,^10^ which described a comparison of the final trial conclusions, total number of patients enrolled, total number of patients allocated to the best performing arm, and trial duration. The number of patients allocated to the best performing arm was included because the SHINE trial included response adaptive randomization, but the bayesian alternative did not. However, the bayesian alternative design used the actual patient data in the re-execution and therefore the actual patient treatment arm assignments. This is a practical limitation of the re-execution that precludes any differences among trial designs on this metric. Comparisons of secondary end points were not prespecified but are included.

All computations for the alternative design were performed in R (R Foundation for Statistical Computing). The code is available at the Deep Blue institutional repository hosted by the University of Michigan Library.^25^

## Results

### SHINE Trial Conduct as Enrolled

The blinded sample size re-estimation did not result in an increase in the study’s sample size, and the study proceeded with the planned interim analyses. A summary of the prespecified boundaries and the observed interim analysis results is given in Table 1. The SHINE trial crossed its prespecified futility boundary at its third interim analysis with 936 patients randomized and 900 consecutively randomized patients completing the 90-day follow-up period. The adjusted estimate of the treatment effect favored intensive glucose control by 0.1%. However, the Data Safety and Monitoring Board (DSMB) did not recommend that the trial stop at this interim, and the trial continued. The prespecified futility boundary was crossed again at the fourth interim analysis with 1137 patients randomized and 1100 consecutive patients included in the analysis. At this interim, the DSMB recommended that the trial stop for futility. The final total sample size of SHINE was 1151 patients. The final primary analysis result showed that 123 of 570 patients (21.6%) in the control arm and 119 of 581 patients (20.5%) in the treatment arm had a favorable 90-day outcome.

**Table 1.** Stroke Hyperglycemia Insulin Network Effort Study Frequentist Interim Analyses and Boundaries

No. of patients enrolled	No. of patients analyzed	Stopping boundaries				No. of patients with 90-d outcome in analysis population	Favorable outcome, unadjusted, No./total No. (%)		Adjusted risk difference, %^a^	*P* value
		Efficacy		Futility			Intensive	Standard		
		*z* Value	*P* value	*z* Value	*P* value					
579	500	2.97	.003	0.06	.95	482	68/254 (26.8)	53/228 (23.3)	2.8	.13
771	700	2.86	.004	0.13	.90	677	83/344 (24.1)	78/333 (23.4)	0.9	.56
936	900	2.65	.008	0.45	.65	869	97/444 (21.9)	97/425 (22.8)	0.1	.96
1137	1100	2.42	.02	1.05	.29	1061	115/540 (21.3)	115/521 (22.1)	−0.1	.92

^a^ With multiple imputation for missing outcome data.

### SHINE Trial Conduct Under Alternative Design

Although the SHINE trial crossed its futility boundary with 936 patients enrolled, the bayesian alternative design crossed the futility boundary at its fourth interim analysis with 800 patients randomized and 715 patients completing the trial for the primary end point. A summary of the results of each of the bayesian alternative design interim analyses is provided in Table 2.

**Table 2.** Bayesian Alternative Design Interim Analyses

No. of patients enrolled	No. of patients with 90-d outcome	Good outcome proportion, %		Predictive probability of trial success, %	
		Treatment	Control	Current sample size success stopping bound >99%	Maximum sample size futility stopping bound <5%
498	432	26.6	24.6	<1	21.1
579	515	25.7	22.3	<1	39.7
700	621	25.1	23.4	<1	15.6
800	715	23.1	22.80	<1	2.0

The Figure shows the predictive distribution of the proportion of patients who experienced a favorable outcome with the currently randomized patients as well as at the maximum sample size of 1400 patients. Considering the similarity of the observed 90-day outcomes, the predictive probability of success at the current sample size was essentially 0%, and the predictive probability of success at the maximum sample size was 2%. This latter quantity was less than the 5% required to stop the trial for futility. Results were not sensitive to the longitudinal model that imputed 90-day outcomes for patients only observed to be complete through 90 days. Repeating the alternative design execution without the longitudinal model resulted in the trial stopping for futility at the same interim analysis.

**Figure.**
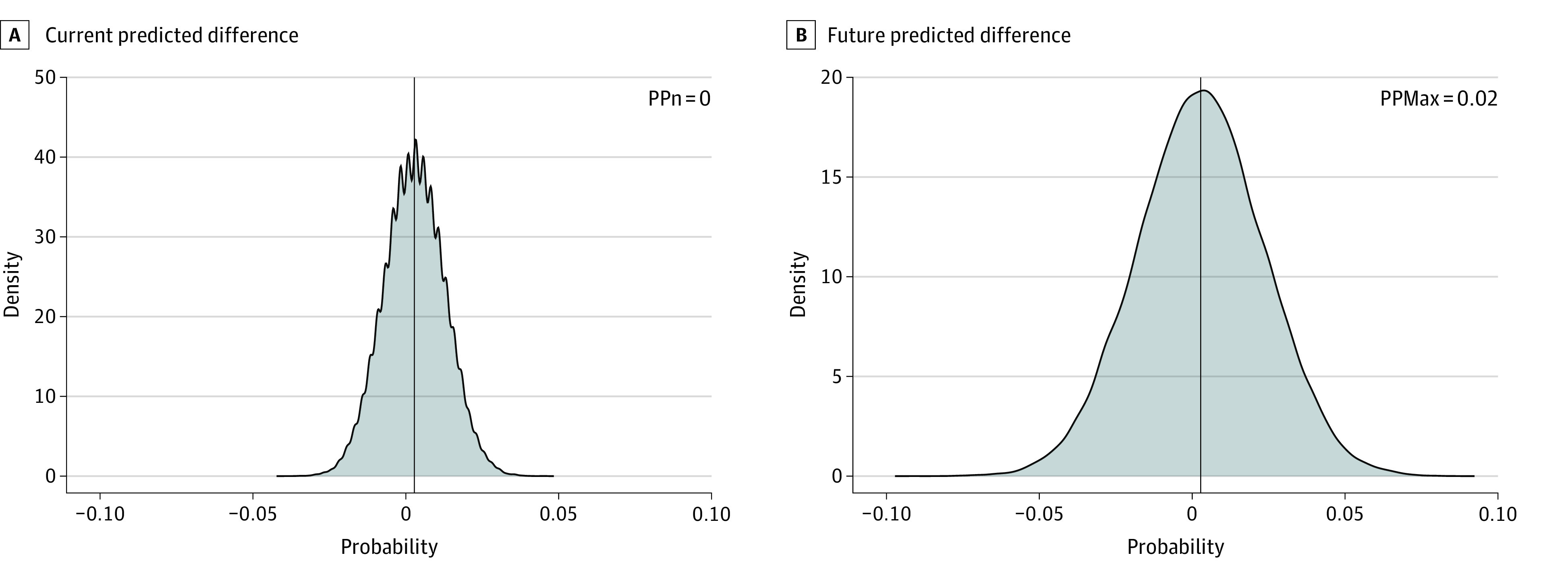
Density Plots for the Posterior Probability of Superiority of Treatment at the Current Sample Size (PPn) and at the Design-Based Maximum Sample Size (PPmax) Values to the left of center favor treatment; values to the right favor the control.

Table 3 gives the results of the primary and key secondary outcomes at the time each trial crossed their futility boundary, not at the time each trial stopped enrollment, because the DSMB decision could not be recreated for the alternative design. Results are summarized with frequentist statistics, including 95% CIs for comparability. Estimates vary slightly, but overall inference is similar between the 2 trial designs across all outcomes.

**Table 3.** Trial Outcomes Based on Design-Based Stopping

Outcome	SHINE frequentist (n = 936)		Shadow SHINE (n = 800)	
	Intensive (n = 483)	Standard (n = 453)	Intensive (n = 408)	Standard (n = 392)
**Primary efficacy outcome**				
Favorable, No. (%)^a^	102 (21.1)	102 (22.5)	89 (21.8)	89 (22.7)
Missing, No. (%)^b^	18 (3.7)	12 (2.7)	14 (3.4)	11 (2.8)
Risk difference, % (95% CI)				
Unadjusted	−1.4 (−6.7 to 3.9)		−0.9 (−6.7 to 4.9)	
Adjusted^c^	−0.1 (−2.6 to 2.5)		0.3 (−2.5 to 3.0)	
*P* value for adjusted analysis	.97		.86	
**Secondary efficacy outcome**				
Favorable NIHSS score (0 or 1), No./total No. (%)	125/293 (42.7)	144/306 (47.1)	109/247 (44.1)	131/270 (48.5)
Unadjusted risk difference, % (95% CI)	−4.4 (−12.4 to 3.6)		−4.4 (−13.0 to 4.2)	
Favorable Barthel Index (range, 95-100), No./total No. (%)	222/408 (54.4)	218/387 (56.3)	193/344 (56.1)	194/336 (57.7)
Unadjusted risk difference, % (95% CI)	−1.9 (−8.8 to 5.0)		−1.6 (−9.1 to 5.8)	
SSQOL				
No. of patients	366	353	312	304
Median (IQR) score	3.73 (2.86 to 4.39)	3.70 (3.07 to 4.46)	3.71 (2.84 to 4.39)	3.73 (3.06 to 4.50)
Difference, medians (95% CI)	0.03 (−0.18 to 0.23)		−0.02 (−0.23 to 0.23)	
**Primary safety outcomes**				
Deaths, No. (%)	44 (9.1)	49 (10.8)	37 (9.1)	41 (10.5)
Risk difference, % (95% CI)	−1.7 (−5.6 to 2.1)		−1.4 (−5.5 to 2.7)	
Severe hypoglycemia (glucose <40 mg/dL [<2.22 mmol/L]), No. (%)	12 (2.5)	0	10 (2.5)	0
Risk difference, % (95% CI)	2.5 (1.1 to 3.9)		2.5 (1.0 to 4.0)	

Abbreviations: NIHSS, National Institutes of Health Stroke Scale; SHINE, Stroke Hyperglycemia Insulin Network Effort; SSQOL, Stroke Specific Quality of Life.

^a^ Favorable for the primary efficacy outcome is defined as a modified Rankin scale (mRS) score of 0 in patients with mild stroke (baseline NIHSS score, 3-7), mRS score of 0 to 1 in patients with moderate stroke (baseline NIHSS score, 8-14), and mRS score of 0 to 2 in patients with severe stroke (baseline NIHSS score, 15-22).

^b^ The analysis for the primary outcome included missing data imputed by a multiple-imputation method.

^c^ The adjusted risk difference for the primary outcome was adjusted for baseline stroke severity (NIHSS scores of 3-7 [mild], 8-14 [moderate], and 15-22 [severe]) and thrombolysis use (yes or no).

## Discussion

Both trial designs reached the same conclusion of futility, but the bayesian alternative design reached that conclusion earlier in participant accrual.^9^ Operating characteristics estimated before trial conduct using numerical simulation under the null hypothesis of no treatment benefit showed that the SHINE trial had an expected sample size of 1039 and that the bayesian alternative had an expected sample size of approximately 750.^10^ The implemented frequentist SHINE design first crossed its futility boundary with 936 patients randomized, and the alternative design crossed its futility boundary with 800 patients randomized. The difference is driven by the enrollment rate and the implemented SHINE design needing to wait until the patients reached the 90-day follow up. Given the data observed during the actual trial, it was more likely than not that both trial designs would stop for futility. Under the null, the SHINE trial had a 73% probability of early futility, and the alternative design had a 91% probability of early futility.^10^

One aspect of the trial execution not reflected with this virtual execution of the alternative design is the DSMB review of the interim analyses and recommendations to continue accrual or stop for futility. In this trial, the stakeholders were interested in providing a definitive answer to the acute stroke community. As such, there was a strong desire to ensure that residual uncertainty was minimized. Examples include the possibility that some patients with acute stroke (mild or severe) might benefit from the intensive glucose management strategy on subgroup analyses. One may speculate that a DSMB would have the same concerns with the bayesian design, particularly if the design suggested stopping even earlier during the trial, but the futility stopping boundaries in the bayesian design were considered to be binding. Overruling the futility stopping in this context could inflate the overall type I error rate of the trial.

Concerns regarding bayesian adaptive design in confirmatory trials include the operational complexity of the frequent interim analyses, limited experience with bayesian algorithms and associated discomfort with how they may perform, and the scientific validity of the decision-making. Bayesian adaptive designs do not necessarily have convention to guide their implementation in the way that frequentist group–sequential trials do. Bayesian statistics can be applied in clinical trials with more flexibility. However, the bayesian alternative design for SHINE still conformed to the standard frequentist hypothesis testing levels of the type I error rate and power, as recommended by regulatory authorities.^26^ The bayesian alternative design of the SHINE trial had more interim analyses, and it could have proposed interims even more frequently. Looking more frequently, as often as after every patient in the extreme, would maximize the probability of stopping the trial early and would minimize the expected sample size because the exact tipping point in the accumulating data that would stop the trial would be identified. However, the benefits of more frequent interim analyses have tradeoffs with decision-making error rates and operational burden. Interim analyses very early in the trial risk incorrect decisions based on spurious results from small samples. More interim analyses not only increase the chances of early stopping but also increase the chances of an incorrect early stop. Frequent futility interim analyses risk decreasing the trial’s power through false-negative conclusions, and frequent interim analyses for success risk increasing type I error rates with a false-positive conclusion. The operational burden of frequent interim analyses includes the trial staffing and allocation of time to monitoring, analyzing, and presenting trial data. As the interim analyses become more frequent, the amount of new data accumulated at each interim decreases. Staff resources are diverted to activities that are increasingly unlikely to provide new and actionable trial results. We advocate that for any proposed design, all possible adaptations are prespecified and that trial stakeholders make design decisions with an understanding of the corresponding operating characteristics, including the expected sample size, the probability of success under the null hypothesis (type I error), and the probability of success under the alternative hypothesis (power).

Regarding the longitudinal modeling used in the bayesian design, some investigators equate bayesian models with informative prior analyses. The goal of this method in the bayesian alternative was to fit longitudinal models that would be driven by the trial data alone to best meet the need of predicting trial success and failure. It is beyond the scope of the current investigation to discuss whether available frequentist models could be used for this form of within-trial decision-making; however, it is notable that they usually are not. Although traditionally only participants with final outcomes contribute to frequentist interim analyses, the partial information from early outcomes was used to inform the bayesian interim analysis. These outcomes can be potentially informative if the uncertainty within it is properly accounted for and understood.

This example shows that it was feasible to collect and freeze the data necessary to conduct the more frequent interim analyses. Although not performed in this virtual execution, the reporting to the DSMB of the interim analysis results can usually be accomplished in a matter of several business days after the interim milestones are achieved with proper systems in place. This example also illustrates throughout the interim analyses how the bayesian algorithm reacted to the observed data and the uncertainty in the data, yet was collected in the same manner and resulted in the same scientific conclusions as the group sequential SHINE trial. This conclusion was reached more efficiently in terms of sample size, was driven by the more frequent interim analyses, and therefore provided a greater opportunity for early stopping. There are multiple examples during COVID-19 showing that rapid, high-quality interim analyses can be conducted in prespecified bayesian adaptive trials.^27^

The group sequential design and the bayesian Goldilocks sample size algorithm are similar. Both techniques aim to limit patient resources in the case of overwhelming efficacy or in the case of futility in the observed data. It is expected that this head-to-head comparison of the 2 approaches resulted in the same trial conclusion, especially considering that the observed trial data did not show a difference between the treatment strategies. As familiarity with the design and operationalization of bayesian adaptive designs increases, we hope to see more innovative features incorporated into confirmatory trials.

### Limitations

This work has some limitations. This is a single example based on a single data set that illustrates the potential of bayesian techniques in confirmatory trials. Although such examples are useful and rare, this study does not generalize that the bayesian approach will always be superior. This work has other important limitations. First, this was a shadow trial conducted post hoc to assess the use of actual data that would have been available at each interim analysis. In practice, however, sufficient resources do not exist to run multiple, concurrent clinical trials to assess differences in designs. Even if that had occurred in this study, the common practice of DSMBs would have likely prompted the cessation of the parallel trial. Second, the trial studied was relatively simple—a 2-group comparison with a binary primary end point. Similar prospective comparisons of more complicated designs, such as those studying multiple doses or having multiple stages, would be more challenging and likely could not use the exact sequence of enrollments and timings of outcomes. Third, although ending a trial early for futility when results are unambiguous is beneficial for the patients and clinical community and can reduce the financial cost, the current method of submitting, reviewing, and issuing grants to researchers is not as well suited to this; the value proposition for a pharmaceutical company bearing all of the investment is clearer.^28^ Fourth, this study was unique among publicly funded US clinical trials in that it had substantial time and monetary resources dedicated to trial design and simulation through the ADAPT-IT project. Although not a limitation of this study per se, it represents a limitation of the current paradigm for proposing, planning, and designing NIH-funded clinical trials in the US.

## Conclusions

The 2 possible SHINE designs performed similarly when exposed to the actual patient responses observed in the trial. The bayesian design reached a conclusion of futility earlier, consistent with the operating characteristics of the null scenario simulated before starting the trial and in part because of the bayesian interim analyses also incorporating incomplete data through a longitudinal model. Trialists should consider developing, simulating, publishing, and assessing counterfactual designs in parallel with actual study conduct to gain additional knowledge and expand understanding of more innovative randomized clinical trials.
